# Impact of Q-Griffithsin anti-HIV microbicide gel in non-human primates: *In situ* analyses of epithelial and immune cell markers in rectal mucosa

**DOI:** 10.1038/s41598-019-54493-4

**Published:** 2019-12-02

**Authors:** Gökçe Günaydın, Gabriella Edfeldt, David A. Garber, Muhammad Asghar, Laura Noȅl-Romas, Adam Burgener, Carolina Wählby, Lin Wang, Lisa C. Rohan, Patricia Guenthner, James Mitchell, Nobuyuki Matoba, Janet M. McNicholl, Kenneth E. Palmer, Annelie Tjernlund, Kristina Broliden

**Affiliations:** 1 Department of Medicine Solna, Division of Infectious Diseases, Center for Molecular Medicine, Karolinska Institutet, Karolinska University Hospital, Stockholm, Sweden; 2 Laboratory Branch, Division of HIV/AIDS Prevention, National Centre for HIV/AIDS, Viral Hepatitis, Sexually Transmitted Disease and Tuberculosis Prevention, CDC, Atlanta, USA; 3 National HIV and Retrovirology Labs, JC Wilt Infectious Diseases Research Centre, Public Health Agency of Canada, Winnipeg, Canada; 4 Department of Obstetrics & Gynecology, and Department of Medical Microbiology, University of Manitoba, Manitoba, Canada; 5 Department of Information Technology, Centre for Image Analysis, Uppsala University, Science for Life Laboratory, Uppsala, Sweden; 6 Magee-Womens Research Institute, Pittsburgh, USA; 7 School of Pharmacy, University of Pittsburgh, Pittsburgh, USA; 8 Center for Predictive Medicine, University of Louisville, Louisville, USA; 9 Department of Pharmacology and Toxicology, University of Louisville, Louisville, USA; 10 James Graham Brown Cancer Center, University of Louisville, Louisville, USA

**Keywords:** Imaging the immune system, Mucosal immunology

## Abstract

Natural-product derived lectins can function as potent viral inhibitors with minimal toxicity as shown *in vitro* and in small animal models. We here assessed the effect of rectal application of an anti-HIV lectin-based microbicide Q-Griffithsin (Q-GRFT) in rectal tissue samples from rhesus macaques. E-cadherin^+^ cells, CD4^+^ cells and total mucosal cells were assessed using *in situ* staining combined with a novel customized digital image analysis platform. Variations in cell numbers between baseline, placebo and Q-GRFT treated samples were analyzed using random intercept linear mixed effect models. The frequencies of rectal E-cadherin^+^ cells remained stable despite multiple tissue samplings and Q-GRFT gel (0.1%, 0.3% and 1%, respectively) treatment. Whereas single dose application of Q-GRFT did not affect the frequencies of rectal CD4^+^ cells, multi-dose Q-GRFT caused a small, but significant increase of the frequencies of intra-epithelial CD4^+^ cells (placebo: median 4%; 1% Q-GRFT: median 7%) and of the CD4^+^ lamina propria cells (placebo: median 30%; 0.1–1% Q-GRFT: median 36–39%). The resting time between sampling points were further associated with minor changes in the total and CD4^+^ rectal mucosal cell levels. The results add to general knowledge of *in vivo* evaluation of anti-HIV microbicide application concerning cellular effects in rectal mucosa.

## Introduction

The global HIV epidemic continues to significantly impact lives, in 2017 approximately 1.8 million new cases, 1 million AIDS deaths, and 37 million HIV infected individuals were reported^1^. The main mode of HIV transmission is through sexual contact with a significantly higher transmission rate for receptive and insertive anal versus vaginal sexual intercourse^2–4^. While the vaginal epithelium is multi-stratified, the rectal epithelium is single-layered and thus more vulnerable with underlying HIV target cells easily reachable for penetrating viral particles. Current oral pre-exposure prophylaxis (PrEP) against HIV infection relies on antiretroviral (ARV) drugs. Although PrEP is highly efficient when treatment adherence is sustained^5–8^, the ARVs can accumulate systemically causing long-term health consequences, and resistant viruses may develop if persons become infected while on PrEP. There is a high demand to complement the oral PrEP options with a rectal microbicide to give individuals who practice anal sex an alternative option for HIV prevention^9^. Microbicides for rectal use could potentially be provided in the form of lubricants, creams, fast dissolving rectal tablets, gels, douches or enemas^10^.

The natural lectin Griffithsin (GRFT) is one of the most potent anti-HIV agents reported to date^11^. GRFT combined with Carrageenan (another antiviral agent derived from algae) in a fast dissolving vaginal insert formulation, was recently shown to protect rhesus macaques (RMs) from a high-dose vaginal SHIV challenge^12^. GRFT is here being assessed as a rectal microbicide, as part of the PREVENT (Griffithsin-based rectal microbicides for PREvention of Viral ENTry) program. PREVENT is an integrated preclinical and clinical international program^13^, with a main goal to deliver a comprehensive set of data on the safety and efficacy profile of GRFT. *In vitro* investigation using human cells has previously confirmed GRFT’s s outstanding safety and efficacy profile as a microbicide candidate^14^. GRFT is isolated from a red algae *Griffithsia sp.*^15^, and has shown broad spectrum antiviral activity to HIV, herpes simplex type 2 virus, and hepatitis C virus in mid-picomolar IC_50_ values^11,15–17^. Bulk production of recombinant GRFT was achieved using *Nicotiana benthamiana*, a plant based protein expression system, offering more than 99% purity, and high stability and activity^18,19^. The method was further improved to make GRFT manufacturing cost-effective, with high yields and low endotoxin, making it a favorable microbicide candidate. Native GRFT is prone to oxidation and to overcome this inherent property of the lectin, an engineered form of wild type GRFT has been developed, Q-GRFT (M78Q), with an expected improved oxidation resistance. Gel based dosage forms based on polymers such as Carbopol, not causing any reported epithelial damage and/or inflammation, are potential delivery vehicles for such topical microbicides^20^.

The aim of this study was to evaluate the *in vivo* effect on mucosal cell populations following rectal application of Q-GRFT gel on the rectal mucosa of healthy RMs. Specifically, the effect on the rectal epithelium (E-cadherin^+^ cells) and the frequencies of CD4^+^ HIV target cells and total number of mucosal cells were assessed in a novel fashion by combining *in situ* immunofluorescence staining and digital image analysis.

## Methods

### Animals

Six purpose-bred RMs (*Macaca mulatta*) were used in this study. The RMs were housed at the Center for Disease Control and Prevention (CDC) in Atlanta, GA, USA, in accordance with the Guide for the Care and Use of Laboratory Animals (8^th^ edition) in an AALAC-accredited facility, following the institutional standard operating procedures^21^. All animal experiments were approved by the CDC Institutional Animal Care and Use Committee (CDC IACUC, protocol 2700SMIMONC).

### Study design

**Figure 1:**
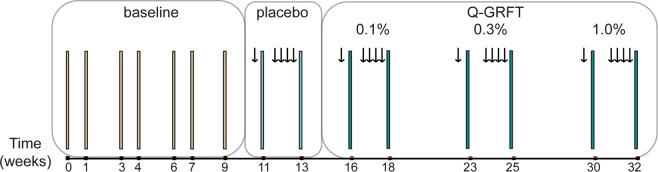
Study outline. Six rhesus macaques were included in the study and two rectal biopsies were collected from each rhesus macaque at each sampling time point. Baseline samples were collected at seven time points during a ten-week period. This was followed by intra-rectal application of the placebo gel in a single-dose setting (4 ml of the gel was applied once and samples were taken on the following day) followed by a multi-dose setting (application of 4 ml placebo gel during four consecutive days prior to sampling on day 5). Thereafter escalating doses of Q-GRFT gel (0.1%, 0.3% and 1% [wt/vol]), in 4 ml per dose, were applied first in the single-dose setting followed by the multi-dose setting as described for the placebo gel. The arrows indicate each gel application and the bars indicate each biopsy sampling point.

The main goal of the study was to evaluate if a microbicide containing different doses of Q-GRFT formulated in a Carbopol gel affected the epithelial barrier integrity and/or the numbers of HIV target cells in the rectal mucosa. Both the placebo (PL)- and the Q-GRFT-gels were Carbopol-formulated, containing Carbopol 974 P (0.75%), methylparaben, propylparaben, sodium hydroxide, disodium ethylenediaminetetraacetic acid and purified water, and applied in 4 ml per dose. The study design and workflow, in terms of rectal biopsy collection- and treatment-schedule are outlined in Fig. 1. Two rectal biopsies were collected from each RM per time point over a time span of 32 weeks. To assess the baseline cell distribution, seven baseline (BL) samples were collected over a period of 10 weeks. Thereafter, each RM received the PL gel intra-rectally, first in a single-dose one day prior to sampling, followed by multi-dose, i.e. during four consecutive days, prior to sampling on the following day. Subsequently, gradually escalating doses of Q-GRFT gel (0.1%, 0.3% and 1% [wt/vol], respectively) were applied intra-rectally, in a single- and multi-dose procedure, respectively. The resting time, i.e. number of days since previous biopsy sampling, varied between 7–14 days throughout the BL sampling, 15–17 days for the PL single and multi-dose application and Q-GRFT multi-dose application, and 20–32 days for the Q-GRFT single-dose application. Throughout the study the number of previous biopsy procedures increases, so at the last sample time point each RM had been exposed to biopsy sampling 15 times. To discern the effects of treatment from potential effects of sampling (number of previous biopsy samplings) and resting time (days since previous biopsy sampling), a linear mixed effects model was used.

### Tissue collection and *in situ* immunofluorescence staining

The rectal biopsies were collected as outlined in Fig. 1. The biopsies were snap frozen in OCT media (Sakura Finetek USA Inc. Torrance, CA) at the CDC, USA. The frozen biopsies were shipped to Karolinska Institutet, Sweden and maintained at −80 °C until sectioning and staining procedures. The cryopreserved rectal biopsies were cut in 8 μm slices using a cryostat, mounted on SuperFrost® Plus Gold slides (Menzel Gläser, Thermo Fischer Scientific, VWR International AB, Spånga, Sweden), air-dried for 1 hr at room temperature (RT), and fixed in 100% methanol for 10 min at RT, which was followed by a wash in PBS.

**Figure 2:**
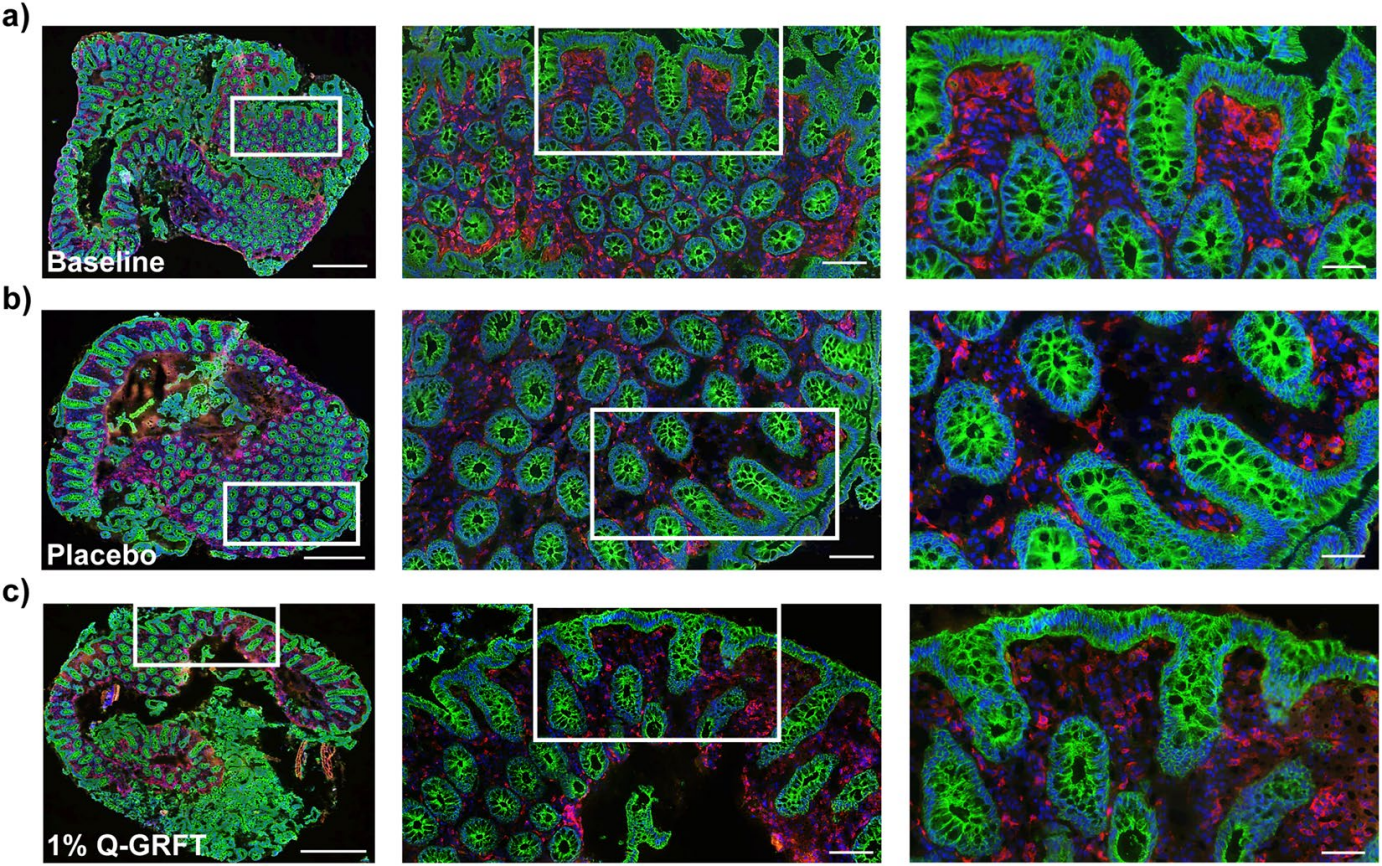
*In situ* staining of E-cadherin and CD4 in rectal tissue. Representative immunofluorescence images of rectal tissue sections from a rhesus macaque stained for E-cadherin (green) and CD4 (red). DAPI (blue) was used as a counterstain for visualization of cell nuclei. The images show staining from a biopsy taken at (**a**) baseline, (**b**) after placebo single-dose and (**c**) after 1% Q-GRFT single-dose. Images in the left column show overview fields of the whole tissue sections (scale bar: 500 μm). Images in the middle column show 20× magnification (scale bar: 100 μm) of the images in the left column. Images in right column show 40× magnification (scale bar: 50 μm) of the regions of interest indicated in the left and middle columns.

The immunofluorescence double staining procedure was performed with E-cadherin and CD4 specific antibodies consecutively, and representative images are shown in Fig. 2. The adherence junction protein E-cadherin was detected using purified monoclonal mouse anti-E-cadherin antibody (610182, clone: 36/E-Cadherin, BD Biosciences, Stockholm, Sweden, 1:50 in antibody diluent, Nordic Biosite AB, Täby, Sweden, BCB-20005), followed by a blocking buffer, composed of donkey serum (2%) and BSA-C (0.1%) diluted in washing buffer (1% HEPES and 0.1% Saponin in PBS), and Alexa Fluor 488 conjugated donkey anti-mouse (highly cross absorbed) (A21202, Lot: 1644644, Invitrogen, Thermo Fischer Scientific, Waltham, MA, 1:200 in blocking buffer) secondary antibody for detection. This was followed by an antigen retrieval step using freshly prepared 0.5% hydrogen peroxide in methanol for 10 min at RT. The CD4^+^ cells were then detected using a purified rabbit anti-CD4 antibody (EPR6855, Abcam, Cambridge, England, 1:200 in antibody diluent), and Alexa Fluor 594 conjugated donkey anti-rabbit (highly cross absorbed, including affinity-purification against mouse immunoglobulins) (A21207, Invitrogen, Thermo Fischer Scientific, 1:400 in antibody diluent) secondary antibody for detection. Tissue sections were counterstained with DAPI (Molecular Probes, Invitrogen, Thermo Fischer Scientific), washed in MilliQ water and thereafter mounted with Fluorescent Mounting Medium (Dako, Carpinteria, CA, USA). Washing buffer was used between each incubation step. Negative controls were included for each tissue section and consisted of incubations in the presence of secondary antibody alone. The stained tissue sections were scanned into digital images using a Pannoramic 250 Flash Slide Scanner (3DHistech Kft., Budapest, Hungary).

### Quantitative image analysis

**Figure 3:**
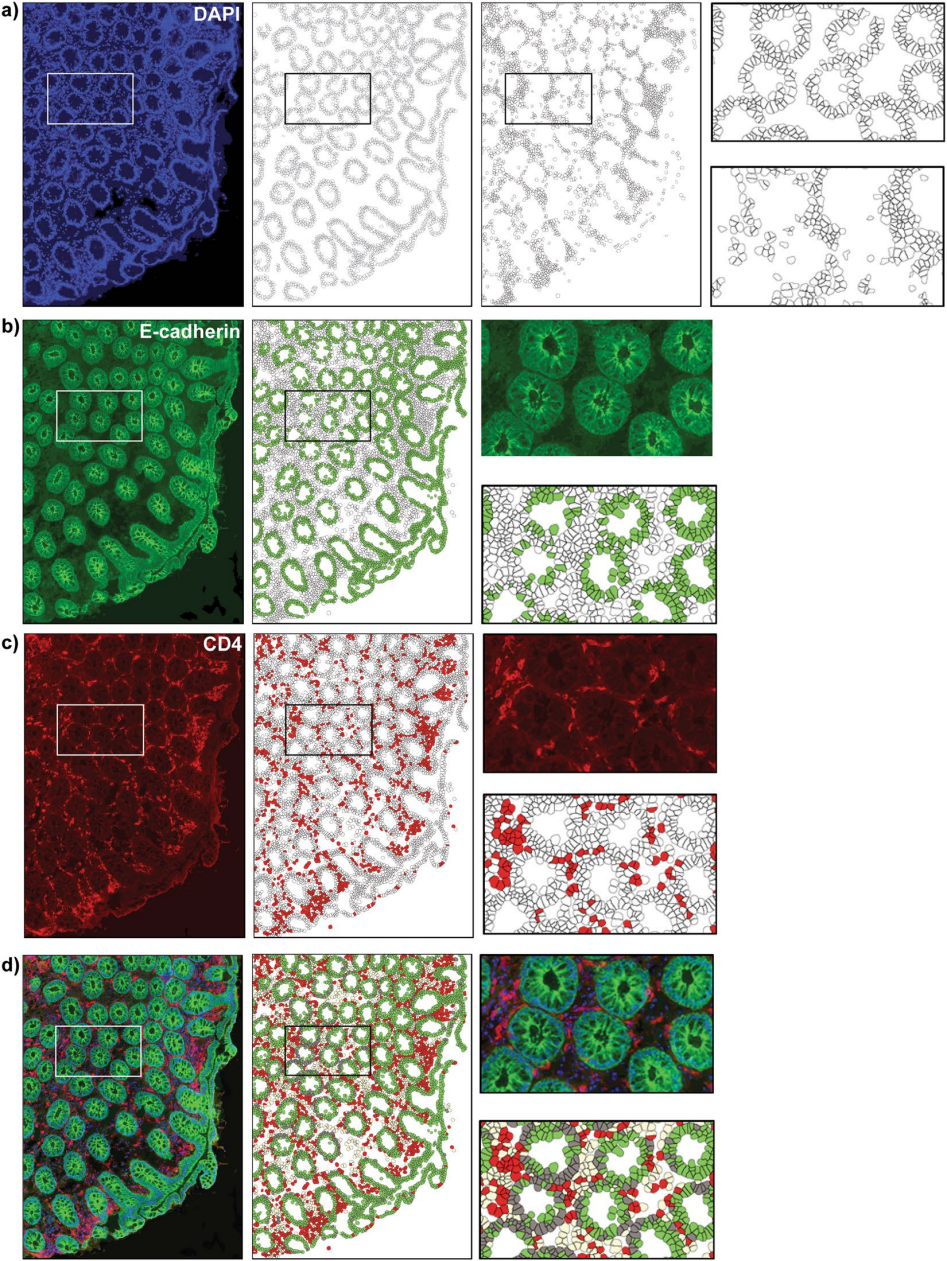
Digital image analysis; work flow. Representative raw and digitalized images of a rectal tissue section stained for E-Cadherin (green), CD4 (red) and DAPI (blue) illustrating the workflow of our customized digital image analysis. (**a**) A raw fluorescent image of DAPI staining (blue, first column) was used for detection of cells, shown as outlines in the second and third column. Cells were detected using CellProfiler, and the software program Ilastik was used to classify cells as either epithelial cells (second column) or lamina propria (LP) cells (third column). The digitalized images in the fourth column are magnified views of the regions indicated in the second and third columns (epithelial cells upper image and LP cells lower image). Likewise, raw images, in the first column, of immunoflourescent staining of (**b**) E-cadherin (green), (**c**) CD4 (red) and (**d**) E-cadherin, CD4 and DAPI. Images in the second column are digitalized images (exported from CellProfiler and colorized in Matlab) representing analysis results of the images in the first column. The raw- and digitalized images in the third column are magnified views of the regions indicated in the first and second column, respectively. The following color codes were used in (**d**) E-cadherin positive epithelial cells in green, E-cadherin negative epithelial cells in dark grey, CD4 positive cells in red and CD4 negative submucosal cells in beige.

Scanned images of the whole tissue sections were exported as.tif files and each image was split in six smaller images to facilitate image workflow. The pixel-based machine learning software Ilastik^22^ was used to classify cell nuclei as either epithelial cells or lamina propria (LP) cells. Areas with background noise were also identified. Together with the Ilastik probability maps, the raw.tif images were uploaded to CellProfiler^23^, where (**i**) tissue area, (**ii**) cell nuclei, (**iii**) LP cells, (**iv**) epithelial cells, (**v**) CD4^+^ cells, (**vi**) E-cadherin^+^ cells, were identified and quantified (Fig. 3.) Areas with background noise were excluded from the analysis and samples with background contamination covering >50% of the tissue area were completely removed from the analysis. Based on the nuclear staining, cell outlines were approximated by enlarging the radius of each nucleus 12 pixels. Since all epithelial cells express E-cadherin to different degrees, we used a fixed threshold where epithelial cells with a mean intensity >0.03 intensity units in the green channel were classified as E-cadherin^+^ cells. This threshold was chosen based on the negative control staining without primary antibody. Classification of CD4^+^ cells was performed in two steps: (**i**) positive stained area in the red channel was identified (by applying a tophat filter followed by an intensity threshold of 0.008 to remove diffuse red fluorescence background), and (**ii**) all cells containing >55 positive red pixels/cell were classified as CD4^+^. The numbers of E-cadherin^+^ cells as well as CD4^+^ cells were normalized by calculating the percentage of positive cells out of total number of respective cell type, in each tissue compartment (i.e. % E-cadherin^+^ cells out of total epithelial cells, and % CD4^+^ cells out of total epithelial cells, i.e. intra-epithelial cells, as well as % CD4^+^ cells out of total LP cells). In addition, the total number of cells per tissue area (cell density), measured as number of cells/mm^2^ tissue, was assessed in each tissue compartment, as an indicator of influx or efflux of cells. Samples lacking, or with low proportion of epithelium (<20% epithelial cells) were excluded from the epithelial cell analyses, and samples with little or no LP (<20% LP cells) were excluded from the LP cell analyses.

### Statistical analysis

All statistical analyses were performed using R (nlme package version 3.1–131.1, longpower package version 1.0–16.1)^24–27^. Variations in the % of E-cadherin^+^ cells, CD4^+^ cells and total cell density between BL, PL and Q-GRFT samples, were analyzed using a random intercept linear mixed effect model (lme) with restricted maximum likelihood (REML) estimations. P values < 0.05 were considered significant. A repeated-measures analysis of variance was used to examine the following fixed effects; (**1**) treatment (PL or Q-GRFT), (**2**) sampling (number of previous biopsy samplings) and (**3**) resting time (days since previous biopsy sampling) on the markers, with two ways interactions term between sampling and resting time. Subject (i.e. RM) was included as a random effect in the model to control for variation between subjects.

Power calculations were performed to estimate the minimum fold change detectable at 80% power.

The lme model was used to investigate if treatment, sampling or resting time, had a significant effect on the cells in the mucosa across all time points. If treatment had a significant effect on the outcome variable, the effect of specific treatments was further investigated by comparing treatment groups one-to-one using the lme model. First, to investigate if the gel itself affected the cells in the mucosa, the PL samples were compared to BL samples. Then, to see if the active compound Q-GRFT added any effect to the gel, the different Q-GRFT concentrations (0.1%, 0.3% and 1% [wt/vol], respectively) were compared to the PL treated samples (single- and multi-dose separately). To verify potential effects of Q-GRFT (compared to PL), each Q-GRFT concentration (0.1%, 0.3% and 1% [wt/vol], respectively) and dose (single and multi-dose) was also compared with the BL samples. Furthermore, to assess variation of the frequencies of E-cadherin^+^ cells, CD4^+^ cells and total mucosal cells over time, the BL samples alone were analyzed using the lme model with sampling and resting time as fixed effects.

Last, all the Q-GRFT treated samples were assessed separately using a lme model with resting time, dose (single – or multi) and concentration (0.1%, 0.3% and 1% [wt/vol], respectively) as fixed effects to elucidate if dosage or the different concentrations of Q-GRFT had an effect on the frequencies of the % of E-cadherin^+^ cells, CD4^+^ cells or the total cell density, respectively.

## Results

### Basic tissue parameters

A total of 180 rectal biopsies were collected from six healthy RMs, including duplicate samples at each of the 15 sampling time points per animal. By creating a customized workflow of digital image analysis, we could objectively evaluate the *in situ* immunofluorescence staining of this large set of rectal tissue biopsies (Fig. 3). Overall, 96% of the collected tissue biopsies had good quality and were included in the image analysis. General tissue characteristics, including total tissue area analyzed (median_all samples_ 3.2 mm^2^, range 0.4–8.0 mm^2^), total number of cells analyzed (median_all samples_ 24,523 cells, range 3,558–63,314 cells), and percentage of epithelial and LP cells out of total number of cells (epithelial: median_all samples_ 51%, range 30–76%; LP: median _all samples_ 48%, range 23–69%) were retrieved (Table 1).

**Table 1:** Tissue characteristics.

Samples	Area mm^2^ Median (Range)	# total cells Median (Range)	% cells in epithelium Median (Range)	% cells in lamina propria Median (Range)
All BL samples	3.3	25, 386	54	46
	(0.4–4.9)	(3, 558–41, 376)	(41–76)	(23–59)
First BL sample, time point 1	3.3	28, 735	49	50
	(1.7–4.7)	(15, 160–41, 376)	(44–58)	(41–56)
Last BL sample, time point 7	3.6	28, 724	57	43
	(2.3–4.9)	(17, 248–33, 644)	(49–67)	(32–51)
BL samples with resting 7 days	3.2	25, 875	52	47
	(0.4–4.5)	(3, 558–40, 720)	(41–69)	(30–59)
BL samples with resting 13/14 days	3.3	24, 473	56	43
	(1.3–4.9)	(10, 063–34, 742)	(44–76)	(23–55)
PL single	4.0	30, 808	48	51
	(1.7–8.0)	(12, 592–63, 314)	(40–54)	(45–59)
PL multi	2.5	18, 019	50	50
	(1.8–4.5)	(11, 845–34, 354)	(42–61)	(38–58)
0.1% Q-GRFT single	3.2	24, 760	50	49
	(2.2–3.6)	(16, 619–27, 991)	(34–59)	(41–66)
0.1% Q-GRFT multi	3.2	26, 369	49	50
	(1.9–4.6)	(14, 467–32, 527)	(45–51)	(48–55)
0.3% Q-GRFT single	3.1	24, 681	49	50
	(2.8–4.9)	(20, 563–39, 118)	(44–54)	(46–56)
0.3% Q-GRFT multi	2.6	20, 014	48	51
	(2.3–5.1)	(17, 764–36, 551)	(30–53)	(47–69)
1% Q-GRFT single	3.0	24, 807	57	43
	(1.7–4.2)	(12, 929–37, 714)	(54–61)	(39–45)
1% Q-GRFT multi	3.6	25, 104	43	57
	(0.8–5.7)	(6, 242–48, 451)	(41–49)	(51–69)
All samples	3.2	24, 523	51	48
	(0.4–8.0)	(3, 558–63, 314)	(30–76)	(23–69)

Abbreviations: BL, baseline; PL placebo; single, single-dose application; multi, multi-dose application. A total of 180 rectal biopsies were collected from six healthy RMs, including duplicate samples at each of the 15 sampling time points per animal. Median and range of total tissue area analyzed, total number of cells analyzed and percentage of epithelial and LP cells out of total number of cells were retrieved from the stained tissue sections using quantitative image analysis.

### Q-GRFT and sampling procedure did not affect the frequencies of E-cadherin^+^ cells

E-cadherin is the core membrane protein of the adherence junction protein family, which together with other junction complex members make up a network of epithelial junction proteins. These proteins contribute to a robust mucosal epithelium that can prevent entry of pathogens^28^. Thus, as a proxy for epithelial integrity, the percentage of E-cadherin^+^ cells out of total epithelial cells was assessed in the rectal samples.

**Figure 4:**
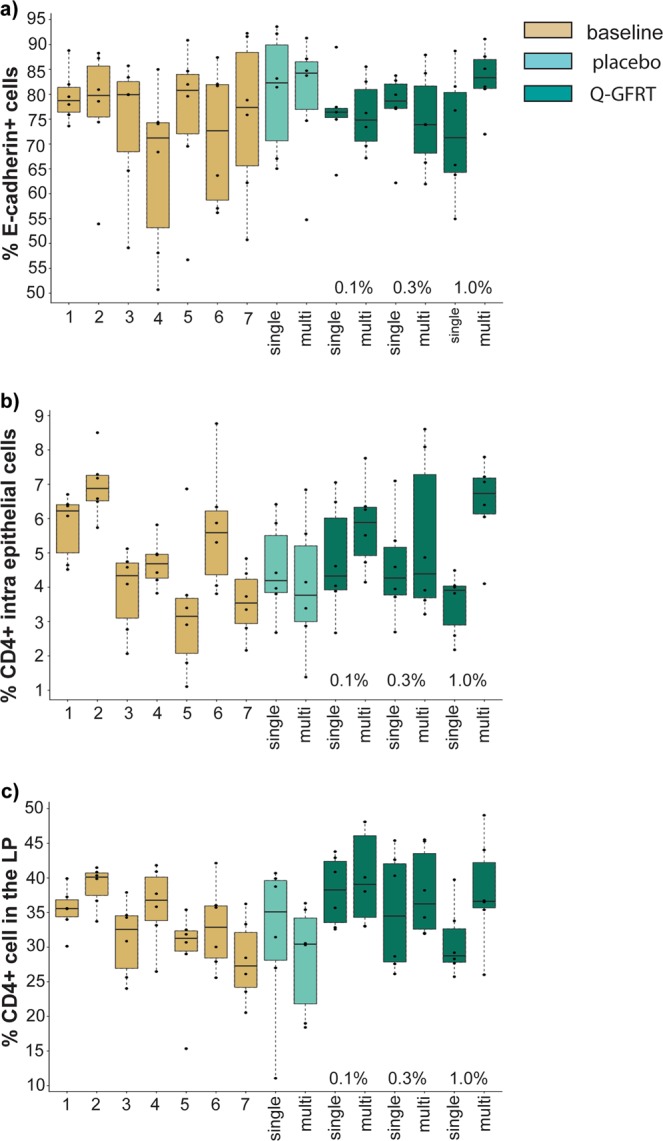
Enumeration of E-cadherin^+^ cells and CD4^+^ cells in the rectal mucosa. The plots show the distribution and median ± interquartile range of (**a**) % of E-cadherin^+^ cells out of total epithelial cells, (**b**) % of CD4^+^intra epithelial cells (i.e. % of CD4^+^ epithelial cells out of total epithelial cells), and **c)** % CD4^+^ Lamina propria (LP) cells (i.e. % of CD4^+^ LP cells out of total LP cells) in rectal samples. *In situ* staining and digital image analysis were used to assess the markers in the rectal biopsies collected during baseline, single and multi-dose placebo treatment as well as after escalating concentrations of Q-GFRT (0.1%, 0.3% and 1% [wt/vol], respectively) in a single- and multi-dose setting. Each circle represents a median value from the two biopsies collected at each time point for each RM.

Immunofluorescence staining combined with digital image analysis was used to measure the frequencies of E-cadherin^+^ cells in the rectal epithelium. The rectal epithelium is covered in mucus causing a dim autofluorescence background. Thus, to allow an unbiased quantification, we analyzed epithelial cells expressing E-cadherin above a mean intensity/cell of 0.03 intensity units. The frequencies of E-cadherin^+^ cells varied between 41–94% with a median of 78% across the experimental setup (i.e. all samples) (Fig. 4a, Table 2). To assess if this variation correlated to Q-GRFT gel treatment, a lme-model was used to analyse the fixed effects of treatment, sampling and resting time. Treatment consisted of either applying the PL gel in a single- or multi-dose, or applying different concentrations of Q-GRFT gel (0.1%, 0.3% or 1% formulation, respectively) in a single- or multi-dose fashion. Treatment did not cause a significant change in the frequencies of E-cadherin^+^ cells in the rectal mucosa. Neither did sampling (number of previous biopsy samplings) or resting time (days since previous biopsy sampling), and no interaction between sampling and resting time was seen (Supplementary Table 1). Thus, Q-GRFT gel treatment did not have a negative impact on the epithelial integrity as defined by E-cadherin^+^ cell frequencies.

**Table 2:** Percentage of E-cadherin^+^ cells and CD4^+^ cells in rectal mucosa.

Samples	% E-cadherin^+^ cells in epithelium Median (Range)	% intra-epithelial CD4^+^ cells Median (Range)	% CD4 cellsin lamina propria Median (Range)
All BL samples	78.7	4.7	34.1
	(40.7–92.2)	(1.1–8.8)	(15.3–42.1)
First BL sample, time point 1	78.7	6.2	35.6
	(73.6–88.8)	(4.5–6.7)	(30.1–39.9)
Last BL sample, time point 7	77.3	3.5	27.3
	(50.7–92.2)	(2.2–4.8)	(20.5–36.3)
BL samples with resting 7 days	74.4	5.8	36.4
	(40.7–88.3)	(3.8–8.8)	(25.6–42.1)
BL samples with resting 13/14 days	79.7	3.6	30.8
	(46.7–92.2)	(1.1–6.9)	(15.3–37.9)
PL single	82.3	4.2	35.1
	(65.0–93.6)	(2.7–6.4)	(11.1–40.7)
PL multi	84.2	3.8	30.4
	(54.8–91.3)	(1.4–6.8)	(18.4–36.3)
0.1% Q-GRFT single	76.4	4.3	38.3
	(63.7–89.5)	(2.7–7.1)	(32.6–43.8)
0.1% Q-GRFT multi	74.8	5.9	39.1
	(67.2–85.5)	(4.1–7.8)	(33.0–48.1)
0.3% Q-GRFT single	78.6	4.3	34.5
	(62.2–83.7)	(2.7–7.1)	(26.1–45.4)
0.3% Q-GRFT multi	73.9	4.4	36.3
	(61.9–87.9)	(3.2–8.6)	(31.9–45.5)
1% Q-GRFT single	71.3	3.9	28.8
	(54.9–88.7)	(2.2–4.5)	(25.7–39.7)
1% Q-GRFT multi	83.3	6.7	36.6
	(72.0–91.1)	(4.1–7.8)	(26.0–49.1)
All samples	78.3	4.6	34.5
	(40.7–93.6)	(1.1–8.8)	(11.1–49.1)

Abbreviations: BL, baseline; PL placebo; single, single-dose application; multi, multi-dose application. Quantitative image analysis were used to assess the % of E-cadherin^+^ cells and CD4^+^ cells in rectal mucosa. Calculations gave the following fold change values with 80% power: 0.4 for E-cadherin^+^ cells, 1.1 for CD4^+^ cells in the epithelium and 0.7 for CD4^+^ cells in the LP.

### Multi-dose Q-GRFT caused a small increase in the frequencies of rectal CD4^+^ cells

Cells positive for the HIV receptor CD4 were next assessed as a proxy for HIV target cells. Digital image analysis revealed that the majority of CD4^+^ cells were localized within the LP of the rectal mucosa, and a few CD4^+^ cells located within the epithelial cell layer (Fig. 3). To assess if treatment affected the frequencies of CD4^+^ cells from these two morphologically and spatially distinct sites, the frequencies of CD4^+^ cells within the epithelium (i.e. CD4^+^ intra-epithelial cells; % CD4^+^ cells out of total epithelial cells) and within the LP (% CD4^+^ cells out of total LP cells) were assessed separately (Fig. 4b,c, Table 2).

The lme model showed that treatment had a significant effect on the frequencies of CD4^+^ cells, both in the epithelium and in the LP (lme_intra-epithelial_: F_8,143_ = 2.2, p = 0.03, lme_LP_: F_8,141_ = 3.1, p = 0.003) (Table 2, Supplementary Table 1). We then investigated which treatment was causing the effect. The gel itself had no effect on the frequencies of CD4^+^ cell in the epithelium or the LP, as shown by comparing the PL (single-dose or multi-dose) to the BL samples (Table 2, Supplementary Table 1). Neither did single-dose application of different concentrations of Q-GRFT gel (0.1%, 0.3% or 1% formulation) differ from applying PL gel in a single-dose, with regard to frequencies of CD4^+^ cells in the epithelium or in the LP (Table 2, Supplementary Table 1). However, the frequencies of intra-epithelial CD4^+^ cells were significantly higher in samples taken after application of the 1% Q-GRFT gel in multi-dose (median 7%), compared to samples taken after application of the multi-dose PL gel (median 4%) (lme_intra-epithelial_: F_1,14_ = 6.8, p = 0.02). Furthermore, in the LP, all samples taken after application of multi-dose Q-GRFT gel (0.1%, 0.3%, 1%), as compared to samples taken after multi-dose PL gel application, showed significantly higher frequencies of CD4^+^ cells (multi-dose PL: median 30%; multi-dose Q-GRFT gel: median 36% – 39%, lme_LP_: for 0.1% Q-GRFT F_1,14_ = 21.5, p = 0.0004; for 0.3% Q-GRFT F_1,16_ = 6.3, p = 0.02; and for 1% Q-GRFT F_1,14_ = 7.3, p = 0.02) (Table 2, Supplementary Table 1).

Comparing Q-GRFT gel vs. all BL samples showed comparable results (as comparing Q-GRFT gel vs. PL samples). I.e. applying the Q-GRFT gel in single-dose had no effect on the frequencies of CD4^+^ cells in the epithelium or in the LP, apart from the single-dose 0.1% Q-GRFT gel which displayed significantly higher frequencies of CD4^+^ cells in LP (BL: median 34%, single-dose 0.1% Q-GRFT: median 38%. lme_LP_: F_1,70_ = 5.0, p = 0.03). Furthermore, 1% multi-dose Q-GRFT gel significantly affected the frequencies of intra-epithelial CD4^+^ cell (BL: median 5%; multi-dose 1% Q-GRFT: median 7%, lme_intra-epithelial_: F_1,71_ = 7.2, p = 0.009), and all concentrations of multi-dose Q-GRFT gel (0.1%, 0.3%, 1%), significantly increased the frequencies of CD4^+^ cell in the LP (BL: median 34%; multi-dose Q-GRFT: median 36% – 39%, lme_LP_: for 0.1% Q-GRFT F_1,68_ = 9.7, p = 0.003, for 0.3% Q-GRFT F_1,70_ = 4.5, p = 0.04, and for 1% Q-GRFT F_1,68_ = 5.7, p = 0.02) (Table 2, Supplementary Table 1).

All the Q-GRFT gel samples were next assessed to tease out if dosage (single or multi), or different concentrations of Q-GRFT gel (0.1%, 0.3% and 1%), affected the frequencies of the CD4^+^ cells. The lme model showed that neither dose nor concentration affected the frequencies of intra-epithelial CD4^+^ cell or the frequencies of CD4^+^ cell in the LP (Supplementary Table 1). A significant interaction (p = 0.02) was seen between dose and concentration for the frequencies of intra-epithelial CD4^+^ cells, but not for CD4^+^ cell in the LP. No interaction was seen between resting time and dose (Supplementary Table 1).

Thus, a slight increase in CD4^+^ cell numbers in the rectal epithelium and LP was detected following multi-dose Q-GRFT gel treatment as compared to PL as well as BL samples.

### Sampling procedures affected the frequencies of CD4^+^ cells in the rectal mucosa

Our lme model was set up to account for potential effects caused by resting time interval and biopsy sampling. The model revealed that the fixed effect of resting time reduced the CD4^+^ cell numbers significantly in both the epithelium and the LP (lme_intra-epithelial_: resting time F_1,143_ = 21.7, p = 0.00001, lme_LP_: resting time F_1,141_ = 9.5, p = 0.003) and that sampling reduced the CD4^+^ cell numbers significantly in the LP (lme_LP_: sampling F_1,141_ = 4.1, p = 0.04). No interaction between resting time and sampling was seen (Supplementary Table 1). To investigate this effect further, the BL samples were assessed alone, to exclude any interference from PL or Q-GRFT gel treatment. Indeed, comparable results were seen for the BL samples, as for taking all samples into account. (lme_intra-epithelial_: resting time F_1,62_ = 18.2, p = 0.0001; lme_LP_: resting time F_1,59_ = 10.5, p = 0.002; sampling F_1,59_ = 4.7, p = 0.03). BL samples collected after a resting period of 7 days had a median of intra-epithelial CD4^+^ cells of 6% and of CD4^+^ cells in the LP of 36%, while samples collected after a resting period of 13/14 days had a median of intra-epithelial CD4^+^ cells of 4% and of CD4^+^ cells in the LP of 31% (Table 2). The CD4^+^ cell frequencies in the LP declined after multiple biopsy samplings from the first BL time point (median of 36% CD4^+^ cells), to the last BL time point (median of 27% CD4^+^ cells in the LP).

Lastly all Q-GFRT gel treated samples were assessed and resting time also affected the frequencies of CD4^+^ cells in both compartments significantly in this set of data (lme_intra-epithelial_: F_1,55_ = 11.2, p = 0.001; lme_LP_: F_1,56_ = 7.8, p = 0.007 (Supplementary Table 1).

These data collectively show a reduced CD4^+^ cell frequency (both in the epithelium and in the LP) in relation to longer resting time as well as reduced CD4^+^ cell numbers in the LP in relation to multiple sampling.

### Resting time but not Q-GFRT treatment affected the total cell numbers in the rectal mucosa

In addition to evaluating CD4^+^ cell frequencies as a proxy for HIV target cells, we assessed if PL and/or Q-GRFT gel treatment affected the total cell density in the tissue (number of cells/mm^2^). Local irritation/inflammation due to sampling and treatment can potentially lead to influx of other cells than CD4^+^ cells into the tissue. The lme model showed that neither PL nor Q-GRFT gel treatment (0.1%, 0.3%, 1% formulation) had any significant effect on the total number of mucosal cells per tissue area, nor when these cells were divided into epithelial or LP compartments (Table 3, Supplementary Table 2). The lme model further showed that resting time caused a small but significant decrease in the total cell density (lme_tot_: resting time F_1,140_ = 4.0, p = 0.05) and in the cell density in the LP (lme_LP_: resting time F_1,141_ = 6.6, p = 0.01), while having no effect on the cell density in the epithelial compartment (Supplementary Table 2). No effect was seen for sampling and there was no interaction between sampling and resting time.

**Table 3:** Total number of mucosal cells in rectal mucosa.

Samples	# cells/mm^2^ in total tissue Median (Range)	# cells/mm^2^ in epithelium Median (Range)	# cells/mm^2^ in lamina propria Median (Range)
All BL samples	8, 183	11, 813	5, 966
	(5, 026–9, 910)	(10, 572–12, 956)	(3, 139–8, 220)
First BL sample, time point 1	8, 775	12, 178	7, 061
	(8, 428–9, 587)	(11, 869–12, 489)	(5,837–7, 740)
Last BL sample, time point 7	7, 386	11, 188	5, 044
	(6, 221–8,983)	(10, 991–11, 928)	(3, 139–7, 206)
BL samples with resting 7 days	8, 338	11, 794	6, 196
	(6, 489–9, 526)	(10, 933–12, 406)	(4, 209–8, 220)
BL samples with resting 13/14 days	7, 509	11, 350	5, 044
	(5, 026–9, 910)	(10, 572–12, 956)	(3, 139–7, 740)
PL single	7, 868	12, 058	5, 506
	(5, 782–9, 545)	(10, 596–12, 778)	(4, 317–8, 080)
PL multi	7, 851	11, 953	5, 124
	(6, 253–8, 141)	(11, 042–12, 608)	(3, 766–6, 532)
0.1% Q-GRFT single	7, 715	11, 931	5, 575
	(7, 055–8, 271)	(11, 491–12, 804)	(4, 866–6, 608)
0.1% Q-GRFT multi	7, 823	11, 942	5, 759
	(7, 142–8, 527)	(11, 868–12, 464)	(5, 356–6, 653)
0.3% Q-GRFT single	7, 763	11, 907	5, 763
	(7, 038–8, 766)	(11, 883–12, 644)	(5, 078–6, 588)
0.3% Q-GRFT multi	7, 607	11, 964	5, 747
	(7,220–8, 206)	(11, 594–12, 270)	(5, 044–6, 485)
1% Q-GRFT single	7, 917	12, 105	5, 228
	(7, 194–9, 064)	(11, 236–12, 489)	(4, 500–6, 594)
1% Q-GRFT multi	7, 617	11, 528	6, 009
	(5, 854–8, 561)	(10, 771–11, 901)	(4, 442–6, 701)
All samples	7, 875	11, 903	5, 708
	(5, 026–9, 910)	(10, 572–12, 956)	(3, 139–8, 220)

Abbreviations: BL, baseline; PL placebo; single, single-dose application; multi, multi-dose application. Quantitative image analysis were used to assess the total numbers of cells within the stained tissue sections.

To avoid any interference from treatment, the BL samples were examined separately, showing that no effect was seen for sampling. Resting time did not have an effect of the total cell density or the cell density in the epithelium, but showed a significant decrease in the cell density in the LP (lme_LP_: resting time F_1,59_ = 4.9, p = 0.03), (Supplementary Table 2). Thus, a longer resting time (14 vs. 7 days) might induce a small decrease of the total cell density.

## Discussion

We here evaluated effects on critical cell populations of a topically applied rectal Q-GRFT microbicide gel in a dose-escalating model in the rectal mucosa of RMs. As a measure of epithelial tissue barrier integrity and HIV target immune cell density, rectal biopsies were assessed for E-cadherin^+^ cells, CD4^+^ cells and total mucosal cell numbers, respectively. Neither the placebo gel nor the different concentrations of Q-GRFT gel affected the frequencies of E-cadherin^+^ cells. Furthermore, neither did the PL gel as such, nor the Q-GRFT gel treatment in a single-dose setting compared to the PL gel cause any change in frequencies of CD4^+^ cells or total mucosal cells in the rectal mucosa. However, Q-GRFT gel treatment at the highest concentration (1%) in the multi-dose setting caused a minor but statistically significant increase of the intra-epithelial CD4^+^ cell numbers. Q-GRFT gel treatment (0.1–1%) in the multi-dose setting also caused a minor but statistically significant increase in the frequencies of CD4^+^ cells in LP. Q-GRFT gel treatment did not affect the total number of mucosal cells; neither in the epithelium nor in the LP, indicating that the increase of CD4^+^ counts may represent upregulation of the CD4 marker rather than an influx of CD4^+^ cells.

The observed increase in CD4^+^ cell numbers was further evaluated for any confounding effects because we noted fluctuations of the frequencies of CD4^+^ cells within the BL samples, prior to any gel treatment (PL or Q-GRFT). Previous studies suggest that not only specific microbicides such as Nonoxynol-9, but also experimental sampling procedures as such, may induce short-term (hrs) rectal epithelial damage in non-human primates^29–31^. Remarkably, in our present study, as the number of BL samplings increased over time, the frequencies of CD4^+^ cells decreased, both in the epithelium and in the LP. This decrease was dependent on the resting time between sampling. The extended resting time in the BL samples, 13 or 14 days, caused a reduction in the frequencies of CD4^+^ cells as compared to the BL samples with 7 days resting time. Furthermore, an extended resting time also caused a reduction in total mucosal cells in the LP. A caveat with this study is that the RMs, in addition to the tissue samples obtained for the present study, were further sampled for rectal fluid and/or swabs, frequently within the first 24 hours, and daily in the multi-dose setting, prior to collection of the biopsies. These additional samplings may be a confounder in our analysis. Thus, multiple sampling and resting time are potential factors that may need to be considered when designing experimental trials. Due to the combined effect of non-homogeneous resting time throughout the study (ranges 7–14 days during BL, and 14–32 days during treatment), and gel treatment, it is challenging to interpret the impact of resting time alone in the entire sample set. Further studies will thus be needed to address a more detailed phenotypic analysis of these CD4^+^ cells, and especially if they represent the activated CD4^+^CCR5^+^ phenotype in the rectal mucosa which has been shown to correlate to SIV acquisition in RMs, in an experimental vaccine model^32^.

The biologic relevance of the minor increase of CD4^+^ cells observed in the rectal mucosa with multiple Q-GRFT dosing is likely to be insignificant. We have previously evaluated a hyperosmolar lubricant for its effect on rectal mucosa and SHIV acquisition^33^. Despite findings of focal infiltrates of mononuclear cells and rectal lavages showing cytokine surges, bleeding and sloughed epithelial cells, we did not observe any increased SHIV acquisition in RMs as compared to placebo treatment. Thus, it is less likely that the minor increases of CD4^+^ cells, together with the stable numbers of E-cadherin^+^ cells and total mucosal cells, in the present study, would be associated with an increased risk of HIV/SIV infection. In the LP, the increase in CD4^+^ cells was slightly less with the higher concentrations than with 0.1% gel, a surprising finding as an inflammatory effect would likely be dose-dependent. However, Q-GRFT was assessed in a narrow range of concentrations (a 10-fold change between the lowest (0.1%) and the highest (1%) concentration). To further understand the biological relevance of the CD4 observations, it would be important to evaluate a wider range of product concentrations, a mechanical control (mock gel), and a positive control known to increase HIV or SIV acquisition *in vivo*, such as imiquimod or Nonoxynol-9. Interestingly, ARV-based microbicides, such as TFV, have been found safe in clinical trials despite numerous mucosal molecular changes including altered epidermal protein expression, up-regulated inflammatory proteins, inhibited wound-healing and T-cell proliferation^34–36^.

The observation in the present study of stable E-cadherin^+^ cell counts, and visually intact epithelial linings in the tissue sections, despite PL or Q-GRFT treatment, indicates a non-toxic effect on the rectal epithelium. Moreover, the human and non-human primate colonic mucosa undergoes rapid restitution following acute injuries^30,37^. The timing of sampling in relation to placebo or microbicide application is thus critical since possible effects such as cytokine responses, microbiome changes and subsequent immune activation and influx of cells follow different kinetic patterns. It will thus be interesting to expand these findings to additional HIV target cell and epithelial junction protein markers as well as sampling time points closer in time to application of the product (placebo or microbicide).

Earlier studies have reported the outstanding safety profile of GRFT including *in vitro* models, explant models and small animal models^14,38^. In a pilot study using a rectal gel containing 0.1% (wt/vol) wild-type GRFT (a less oxidation-resistant compound as compared to Q-GFRT) we have also evaluated the effects of single-dose PL and GRFT gel by assessing proteome and microbiome data from rectal samples from RMs^39^. Remarkably, the GRFT microbicide gel, when formulated in either HEC or Carbopol, was not associated with any significant changes in protein levels, but with an increased abundance of two common and beneficial microbial taxa related to application of the HEC-GRFT gel. However, both placebo formulations (HEC and Carbopol) were associated with temporary alterations in expression of proteins involved in proteolysis, and in activation of the immune response and inflammation. Thus, application of topical rectal compounds may induce an inflammatory response.

In conclusion, this study represents a first step in the *in vivo* evaluation of a non ARV-based microbicide product with regards to cellular effects *in situ* in the rectal mucosa. The study highlights that the frequencies of rectal E-cadherin^+^ cells remain stable despite multiple rectal tissue samplings and local Q-GRFT treatment, whereas the application of Q-GRFT treatment (multi-dose) results in minor increases in the frequencies of rectal mucosal CD4^+^ cells. The resting time between sampling points were further associated with minor changes in the total and CD4^+^ rectal mucosal cell levels and needs to be observed in similar future studies. Although statistically significant, the biological relevance of the minor changes in CD4^+^ cell counts for mucosal susceptibility to virus infection is most likely negligible, but needs attention in a future clinical trial. Furthermore, a novel customized digital image analysis workflow was developed for assessment of these cell populations for both rectal epithelium and lamina propria, respectively. Using this image analyses technique, we ultimately aim to build a platform for safety trials in which the number and location of several types of HIV target cells (T cells, dendritic cells and monocytes and their distinct phenotypes) and epithelial junction markers can be assessed. The analysis platform could thus complement traditional methods of safety evaluations by highlighting markers of relevance for HIV transmission. The technique is especially interesting for larger clinical trials since the computerized analysis can handle a large set of samples in an objective and high-throughput manner. Lastly, these results also add to general knowledge of RM rectal mucosal histology and should be relevant for other types of experimental studies in non-human primates.

## Supplementary information

Supplementary Table 1 and 2

## Data Availability

All relevant data are presented within the paper and in the supplementary files and are furthermore available upon request from the corresponding author.
