# Supplementary material for: Impact of Q-Griffithsin anti-HIV microbicide gel in non-human primates: In situ analyses of epithelial and immune cell markers in rectal mucosa
Source: Sci Rep. 2019 Dec 2;9:18120. doi: 10.1038/s41598-019-54493-4 (PMC6889265; doi:10.1038/s41598-019-54493-4)
Supplement: Supplementary file 1 — Supplementary Table 1 and 2 [file 41598_2019_54493_MOESM1_ESM.pdf]

Impact of Q-Griffithsin anti-HIV microbicide gel in non-human primates: *In situ* analyses of epithelial and immune cell markers in rectal mucosa

Gökçe Günaydın[1,11], Gabriella Edfeldt[1,11], David A. Garber[2], Muhammad Asghar[1], Laura Noël-Romas[3,4], Adam Burgener[1,3,4], Carolina Wählby[5], Lin Wang[6], Lisa C. Rohan[6,7], Patricia Guenthner[2], James Mitchell[2], Nobuyuki Matoba[8,9,10], Janet M. McNicholl[2], Kenneth E. Palmer[8,9,10], Annelie Tjernlund[1,11*] and Kristina Broliden[1,11]

Supplementary Table 1. Variations in the % of E-cadherin+ cells and the % of CD4+ cells between BL, PL and Q-GRFT samples, were analysed using a random intercept linear mixed effect model (lme) with restricted maximum likelihood (REML) estimations.

		% E-cadherin^{+} epithelial cells					% intra-epithelial CD4^{+} cells					% CD4^{+} cells in LP				
**Samples**	**Fixed Effects/Factors**	**Est.**	**SE**	**DF**	**F-value**	**p-value**	**Est.**	**SE**	**DF**	**F-value**	**p-value**	**Est.**	**SE**	**DF**	**F-value**	**p-value**
BL vs. ALL	(Intercept)	79.4	15.1	143	1698.2		9.5	2.1	143	252.7		48.9	9.4	141	902.3	
	Treatment				1.4	0.2				2.2	**0.03**				3.1	**0.003**
	Study_ArmPLm	1.7	9.1	143			1.5	1.3	143			4.3	5.5	141		
	Study_ArmPLs	1.3	7.6	143			1.7	1.1	143			5.9	4.6	141		
	Study_ArmQ0.1m	-4.8	12.3	143			3.3	1.7	143			17.8	7.5	141		
	Study_ArmQ0.1s	-15.5	18.5	143			3.2	2.6	143			16.6	11.3	141		
	Study_ArmQ0.3m	-14.8	20.2	143			3.2	2.8	143			17.5	12.3	141		
	Study_ArmQ0.3s	-52.9	53.4	143			4.9	7.5	143			19.6	32.6	141		
	Study_ArmQ1m	-9.0	24.8	143			4.8	3.5	143			20.1	15.1	141		
	Study_ArmQ1s	-68.3	66.9	143			3.4	9.4	143			14.3	40.9	141		
	Resting time	-0.5	1.5	143	1.5	0.2	-0.4	0.2	143	21.7	**0.00001**	-1.1	0.9	141	9.5	**0.003**
	Sampling	-3.1	3.4	143	0.1	0.8	-0.4	0.5	143	2.6	0.1	-1.8	2.1	141	4.1	**0.04**
	Resting time:Sampling	0.3	0.3	143	0.7	0.4	0.0	0.0	143	0.2	0.7	0.1	0.2	141	0.1	0.8
BL vs. PLs	(Intercept)	80.4	15.7	73	299.6		9.5	2.2	73	166.9		48.5	9.5	70	461.4	
	Treatment	1.3	7.6	73	3.0	0.1	1.7	1.1	73	0.03	0.9	6.0	4.6	70	0.2	0.7
	Resting time	-0.6	1.5	73	1.4	0.2	-0.4	0.2	73	19.5	**0.00003**	-1.0	0.9	70	9.3	**0.003**
	Sampling	-3.3	3.4	73	0.1	0.7	-0.4	0.5	73	2.3	0.1	-1.8	2.1	70	4.1	**0.05**
	Resting time:Sampling	0.3	0.3	73	0.8	0.4	0.0	0.0	73	0.2	0.7	0.1	0.2	70	0.1	0.8
BL vs. PLm	(Intercept)	80.4	16.1	72	350.5		9.5	2.3	72	138.6		49.0	8.9	69	626.1	
	Treatment	2.0	9.4	72	3.9	0.1	1.5	1.4	72	0.4	0.5	4.4	5.2	69	1.9	0.2
	Resting time	-0.6	1.5	72	1.3	0.3	-0.4	0.2	72	18.9	**0.00004**	-1.1	0.9	69	10.5	**0.002**
	Sampling	-3.3	3.5	72	0.1	0.7	-0.4	0.5	72	2.2	0.1	-1.9	2.0	69	4.7	**0.03**
	Resting time:Sampling	0.3	0.3	72	0.8	0.4	0.0	0.0	72	0.2	0.7	0.1	0.2	69	0.1	0.7
PLs vs. 0.1% QGRFTs	(Intercept)	80.4	3.5	17	713.9		4.5	0.6	17	82.1		31.5	3.1	17	218.8	
	Treatment	-4.0	4.0	17	1.0	0.3	0.3	0.7	17	0.2	0.7	6.6	4.1	17	2.6	0.1
PLs vs. 0.3% QGRFTs	(Intercept)	80.4	3.5	16	707.4		4.5	0.6	16	74.9		31.5	3.8	16	100.0	
	Treatment	-3.7	3.8	16	0.9	0.4	0.2	0.6	16	0.1	0.8	4.5	3.8	16	1.4	0.2
PLs vs. 1% QGRFTs	(Intercept)	80.4	4.3	17	438.7		4.5	0.5	17	104.4		31.5	3.1	17	142.3	
	Treatment	-8.5	4.5	17	3.5	0.1	-1.0	0.5	17	4.2	0.1	-0.7	3.4	17	0.0	0.8
PLm vs. 0.1% QGRFTm	(Intercept)	80.7	3.7	14	575.0		4.2	0.7	14	64.7		28.7	3.0	14	153.0	
	Treatment	-4.5	3.6	14	1.5	0.2	1.5	0.7	14	4.4	0.1	11.6	2.5	14	21.5	**0.0004**
PLm vs. 0.3% QGRFTm	(Intercept)	80.8	4.2	15	450.9		4.3	0.7	15	101.8		29.2	2.6	16	343.9	
	Treatment	-6.3	4.2	15	2.3	0.2	0.8	0.9	15	0.8	0.4	8.7	3.5	16	6.3	**0.02**
PLm vs. 1% QGRFTm	(Intercept)	80.5	3.9	14	541.3		4.3	0.6	14	144.2		29.0	3.2	14	152.2	
	Treatment	2.6	3.4	14	0.6	0.5	2.3	0.9	14	6.8	**0.02**	9.4	3.5	14	7.3	**0.02**
BL vs. 0.1% QGRFTs	(Intercept)	80.7	16.0	73	360.6		9.6	2.3	73	232.3		49.5	9.0	70	1042.5	
	Treatment	-16.0	19.2	73	0.6	0.4	3.2	2.9	73	0.05	0.8	16.1	10.8	70	5.0	**0.03**
	Resting time	-0.6	1.5	73	1.3	0.3	-0.4	0.2	73	18.1	**0.0001**	-1.1	0.9	70	10.2	**0.002**
	Sampling	-3.3	3.5	73	0.1	0.7	-0.4	0.5	73	2.2	0.1	-2.0	2.0	70	4.6	**0.04**
	Resting time:Sampling	0.3	0.3	73	0.8	0.4	0.0	0.0	73	0.2	0.7	0.1	0.2	70	0.1	0.7
BL vs. 0.3% QGRFTs	(Intercept)	80.9	15.5	72	291.9		9.5	2.2	72	148.6		49.1	9.3	69	842.0	
	Treatment	-56.6	53.1	72	0.2	0.6	4.9	7.9	72	0.0003	1.0	18.5	32.1	69	1.5	0.2
	Resting time	-0.7	1.4	72	1.4	0.2	-0.4	0.2	72	19.6	**0.00003**	-1.1	0.9	69	9.7	**0.003**
	Sampling	-3.4	3.4	72	0.1	0.7	-0.4	0.5	72	2.3	0.1	-1.9	2.1	69	4.2	**0.04**
	Resting time:Sampling	0.3	0.3	72	0.9	0.4	0.0	0.0	72	0.2	0.7	0.1	0.2	69	0.1	0.7
BL vs. 1% QGRFTs	(Intercept)	81.4	15.3	73	244.0		9.6	2.2	73	192.8		48.8	8.5	70	721.1	
	Treatment	-72.9	65.1	73	0.1	0.8	3.3	9.7	73	3.3	0.07	14.5	36.9	70	0.6	0.4
	Resting time	-0.7	1.4	73	1.4	0.2	-0.4	0.2	73	20.6	**0.00002**	-1.0	0.8	70	11.3	**0.001**
	Sampling	-3.5	3.3	73	0.2	0.7	-0.4	0.5	73	2.5	0.1	-1.8	1.9	70	5.1	**0.03**
	Resting time:Sampling	0.3	0.3	73	1.0	0.3	0.0	0.0	73	0.2	0.7	0.1	0.2	70	0.1	0.7
BL vs. 0.1% QGRFTm	(Intercept)	80.7	16.0	71	343.8		9.6	2.3	71	171.3		49.5	8.8	68	1103.5	
	Treatment	-4.2	12.7	71	1.1	0.3	3.3	1.8	71	2.4	0.1	17.7	7.0	68	9.7	**0.003**
	Resting time	-0.6	1.5	71	1.3	0.3	-0.4	0.2	71	19.4	**0.00004**	-1.1	0.9	68	10.7	**0.002**
	Sampling	-3.3	3.5	71	0.1	0.7	-0.4	0.5	71	2.3	0.1	-2.0	2.0	68	4.7	**0.03**
	Resting time:Sampling	0.3	0.3	71	0.8	0.4	0.0	0.0	71	0.2	0.7	0.1	0.2	68	0.2	0.7
BL vs. 0.3% QGRFTm	(Intercept)	80.4	16.5	72	355.0		9.6	2.4	72	300.3		49.8	9.0	70	1402.5	
	Treatment	-14.7	21.5	72	0.3	0.6	3.2	3.2	72	0.4	0.5	16.7	11.9	70	4.5	**0.04**
	Resting time	-0.6	1.6	72	1.3	0.3	-0.4	0.2	72	17.3	**0.0001**	-1.1	0.9	70	10.3	**0.002**
	Sampling	-3.3	3.6	72	0.1	0.7	-0.4	0.5	72	2.1	0.2	-2.0	2.0	70	4.5	**0.04**
	Resting time:Sampling	0.3	0.3	72	0.7	0.4	0.0	0.0	72	0.2	0.7	0.1	0.2	70	0.2	0.7
BL vs. 1% QGRFTm	(Intercept)	80.6	15.9	71	340.6		9.6	2.3	71	211.4		48.7	9.0	68	584.9	
	Treatment	-9.2	25.3	71	5.8	**0.02**	4.8	3.8	71	7.2	**0.009**	20.5	14.3	68	5.7	**0.02**
	Resting time	-0.6	1.5	71	1.4	0.2	-0.4	0.2	71	18.6	**0.0001**	-1.0	0.9	68	10.2	**0.002**
	Sampling	-3.3	3.5	71	0.1	0.7	-0.4	0.5	71	2.2	0.1	-1.8	2.0	68	4.6	**0.03**
	Resting time:Sampling	0.3	0.3	71	0.8	0.4	0.0	0.0	71	0.2	0.7	0.1	0.2	68	0.1	0.8
BL	(Intercept)	81.1	15.7	62	224.4		9.6	2.3	62	171.8		49.1	8.9	59	725.1	
	Resting time	-3.4	3.4	62	1.4	0.2	-0.4	0.5	62	18.2	**0.0001**	-1.9	2.0	59	10.5	**0.002**
	Sampling	-0.7	1.4	62	0.1	0.7	-0.4	0.2	62	2.2	0.1	-1.1	0.9	59	4.7	**0.03**
	Resting time:Sampling	0.3	0.3	62	0.9	0.3	0.0	0.0	62	0.2	0.7	0.1	0.2	59	0.1	0.7
All QGRFT	(Intercept)	73.9	10.2	55	1779.7		4.6	1.8	55	344.1		40.3	8.1	56	1344.0	
	Resting time	0.2	0.4	55	1.8	0.2	0.0	0.1	55	11.2	**0.001**	-0.1	0.3	56	7.8	**0.007**
	Dose	36.1	42.4	55	0.1	0.7	8.5	7.3	55	1.2	0.3	24.9	33.1	56	0.0	0.9
	Concentration	-7.1	6.1	55	0.6	0.5	-1.6	1.0	55	0.1	0.7	-7.2	4.9	56	0.8	0.4
	Resting time:Dose	-2.5	2.6	55	0.1	0.7	-0.5	0.5	55	0.04	0.8	-1.6	2.1	56	0.0	0.9
	Dose:Concentration	19.9	8.9	55	5.0	**0.03**	3.7	1.5	55	5.8	**0.02**	9.2	7.0	56	1.7	0.2

Abbreviations: BL, baseline; PL, placebo; s, single-dose application; m, multi-dose application.

Supplementary Table 2. Variations in total number of cells between BL, PL and Q-GRFT samples were analysed using a random intercept linear mixed effect model (lme) with restricted maximum likelihood (REML) estimations.

		# cells / mm^2 in total tissue					# cells / mm^2 in epithelium					# cells / mm^2 in LP				
**Samples**	**Fixed Effects/Factors**	**Est.**	**SE**	**DF**	**F-value**	**p-value**	**Est.**	**SE**	**DF**	**F-value**	**p-value**	**Est.**	**SE**	**DF**	**F-value**	**p-value**
BL vs. ALL	(Intercept)	9591	1440	140	1396.4		12537	975	143	17685.0		6673	1471	141	557.1	
	Treatment				0.6	0.8				0.8	0.6				0.3	1.0
	Study_ArmPLm	270	838	140			389	586	143			1058	853	141		
	Study_ArmPLs	539	697	140			439	488	143			1351	710	141		
	Study_ArmQ0.1m	1079	1137	140			716	793	143			2123	1158	141		
	Study_ArmQ0.1s	916	1720	140			561	1198	143			2701	1751	141		
	Study_ArmQ0.3m	1104	1877	140			496	1305	143			3048	1908	141		
	Study_ArmQ0.3s	1912	4962	140			381	3448	143			6751	5053	141		
	Study_ArmQ1m	1021	2302	140			62	1599	143			3588	2344	141		
	Study_ArmQ1s	2075	6228	140			115	4322	143			7448	6342	141		
	Resting time	-97	138	140	4.0	**0.05**	-60	94	143	1.4	0.2	-15	140	141	6.6	**0.01**
	Sampling	-183	317	140	2.2	0.1	-122	219	143	0.7	0.4	55	323	141	2.6	0.1
	Resting time:Sampling	5	29	140	0.02	0.9	7	20	143	0.1	0.7	-20	30	141	0.4	0.5
BL vs. PLs	(Intercept)	9617	1681	70	551.3		12516	1162	73	5125.9		6694	1703	70	254.2	
	Treatment	541	807	70	0.2	0.6	440	580	73	0.4	0.5	1350	815	70	0.01	0.9
	Resting time	-99	160	70	3.1	0.1	-58	112	73	0.9	0.3	-19	161	70	5.1	**0.03**
	Sampling	-187	368	70	1.6	0.2	-118	260	73	0.5	0.5	53	371	70	1.9	0.2
	Resting time:Sampling	5	34	70	0.02	0.9	6	24	73	0.1	0.8	-19	34	70	0.3	0.6
BL vs. PLm	(Intercept)	9629	1690	69	739.2		12546	1169	72	7893.2		6690	1684	69	286.2	
	Treatment	266	981	69	1.6	0.2	391	703	72	0.2	0.7	1045	972	69	1.1	0.3
	Resting time	-100	162	69	3.0	0.1	-60	113	72	1.0	0.3	-17	160	69	5.2	**0.03**
	Sampling	-190	372	69	1.6	0.2	-124	262	72	0.5	0.5	53	368	69	2.0	0.2
	Resting time:Sampling	5	35	69	0.02	0.9	7	24	72	0.1	0.8	-20	34	69	0.3	0.6
PLs vs. 0.1% QGRFTs	(Intercept)	7760	380	17	658.0		11899	242	17	3997.1		5845	399	17	414.6	
	Treatment	-111	466	17	0.1	0.8	110	303	17	0.1	0.7	-210	564	17	0.1	0.7
PLs vs. 0.3% QGRFTs	(Intercept)	7760	404	16	537.9		11899	250	16	2840.1		5845	425	16	288.7	
	Treatment	155	467	16	0.1	0.7	177	233	16	0.6	0.5	58	521	16	0.01	0.9
PLs vs. 1% QGRFTs	(Intercept)	7760	417	17	479.3		11899	260	17	2879.9		5845	451	17	222.4	
	Treatment	277	417	17	0.4	0.5	104	267	17	0.2	0.7	-365	488	17	0.6	0.5
PLm vs. 0.1% QGRFTm	(Intercept)	7391	290	14	1334.8		11832	189	14	7659.0		5328	344	14	394.6	
	Treatment	597	421	14	2.0	0.2	281	274	14	1.1	0.3	597	416	14	2.1	0.2
PLm vs. 0.3% QGRFTm	(Intercept)	7391	328	15	1042.7		11832	224	15	5602.5		5338	386	16	290.9	
	Treatment	270	461	15	0.3	0.6	60	317	15	0.0	0.9	435	391	16	1.2	0.3
PLm vs. 1% QGRFTm	(Intercept)	7398	419	14	490.7		11832	242	14	4418.6		5331	417	14	288.1	
	Treatment	51	532	14	0.01	0.9	-372	351	14	1.1	0.3	484	544	14	0.8	0.4
BL vs. 0.1% QGRFTs	(Intercept)	9605	1634	70	789.7		12547	1149	73	9139.9		6665	1677	70	370.5	
	Treatment	908	1947	70	0.5	0.5	556	1415	73	1.0	0.3	2703	1996	70	0.1	0.7
	Resting time	-99	156	70	3.2	0.1	-60	111	73	1.0	0.3	-16	160	70	5.1	**0.03**
	Sampling	-185	359	70	1.7	0.2	-124	258	73	0.5	0.5	57	368	70	1.9	0.2
	Resting time:Sampling	5	33	70	0.02	0.9	7	24	73	0.1	0.8	-20	34	70	0.3	0.6
BL vs. 0.3% QGRFTs	(Intercept)	9595	1643	69	770.8		12540	1132	72	8067.4		6671	1652	69	336.3	
	Treatment	1905	5645	69	0.002	1.0	379	4005	72	1.5	0.2	6753	5659	69	0.1	0.7
	Resting time	-98	157	69	3.2	0.1	-60	109	72	1.0	0.3	-17	157	69	5.3	**0.02**
	Sampling	-183	361	69	1.7	0.2	-123	254	72	0.5	0.5	57	362	69	2.0	0.2
	Resting time:Sampling	5	34	69	0.02	0.9	7	24	72	0.1	0.8	-20	34	69	0.3	0.6
BL vs. 1% QGRFTs	(Intercept)	9580	1626	70	755.3		12548	1147	73	9343.7		6652	1642	70	309.5	
	Treatment	2117	7008	70	0.02	0.9	90	5091	73	1.0	0.3	7513	7050	70	0.5	0.5
	Resting time	-96	155	70	3.2	0.1	-60	111	73	1.0	0.3	-15	156	70	5.4	**0.02**
	Sampling	-180	357	70	1.7	0.2	-124	258	73	0.5	0.5	60	359	70	2.0	0.2
	Resting time:Sampling	4	33	70	0.02	0.9	7	24	73	0.1	0.8	-20	33	70	0.4	0.5
BL vs. 0.1% QGRFTm	(Intercept)	9629	1630	68	756.4		12549	1148	71	9360.1		6710	1632	68	325.7	
	Treatment	1052	1282	68	0.004	1.0	718	935	71	1.5	0.2	2085	1279	68	0.02	0.9
	Resting time	-101	156	68	3.3	0.1	-60	111	71	1.0	0.3	-20	155	68	5.6	**0.02**
	Sampling	-189	358	68	1.7	0.2	-125	258	71	0.5	0.5	50	357	68	2.0	0.2
	Resting time:Sampling	5	33	68	0.02	0.9	7	24	71	0.1	0.8	-19	33	68	0.3	0.6
BL vs. 0.3% QGRFTm	(Intercept)	9618	1662	69	809.4		12553	1167	72	9923.7		6700	1648	70	347.4	
	Treatment	1081	2164	69	0.4	0.5	487	1565	72	0.4	0.6	3023	2132	70	0.003	1.0
	Resting time	-99	159	69	3.1	0.1	-61	113	72	1.0	0.3	-18	157	70	5.4	**0.02**
	Sampling	-188	366	69	1.6	0.2	-125	262	72	0.5	0.5	51	361	70	2.0	0.2
	Resting time:Sampling	5	34	69	0.02	0.9	7	24	72	0.1	0.8	-19	34	70	0.3	0.6
BL vs. 1% QGRFTm	(Intercept)	9632	1739	68	874.2		12553	1183	71	9452.3		6706	1734	68	389.1	
	Treatment	981	2783	68	1.3	0.3	73	1944	71	0.4	0.5	3530	2767	68	0.01	0.9
	Resting time	-101	167	68	2.8	0.1	-61	114	71	0.9	0.3	-20	166	68	4.8	**0.03**
	Sampling	-190	384	68	1.5	0.2	-125	266	71	0.5	0.5	50	382	68	1.8	0.2
	Resting time:Sampling	5	36	68	0.02	0.9	7	25	71	0.1	0.8	-19	35	68	0.3	0.6
BL	(Intercept)	9621	1730	59	639.4		12547	1212	62	7121.1		6692	1731	59	277.5	
	Resting time	-100	165	59	2.9	0.1	-60	117	62	0.9	0.3	-19	165	59	4.9	**0.03**
	Sampling	-188	380	59	1.5	0.2	-124	272	62	0.5	0.5	53	379	59	1.8	0.2
	Resting time:Sampling	5	35	59	0.02	0.9	7	25	62	0.1	0.8	-20	35	59	0.3	0.6
All QGRFT	(Intercept)	7254	860	55	5227.5		11850	574	55	27548		5031	915	56	1632.3	
	Resting time	19	36	55	1.2	0.3	9	24	55	1.2	0.3	33	38	56	0.3	0.6
	Dose	2789	3586	55	0.1	0.8	1063	2394	55	0.9	0.3	2168	3724	56	0.3	0.6
	Concentration	179	522	55	0.5	0.5	-121	348	55	4.5	**0.04**	-616	553	56	0.6	0.4
	Resting time:Dose	-154	225	55	1.5	0.2	-58	150	55	1.2	0.3	-118	233	56	0.003	1.0
	Dose:Concentration	-468	755	55	0.4	0.5	-496	504	55	1.0	0.3	693	792	56	0.8	0.4

Abbreviations: BL, baseline; PL, placebo; s, single-dose application; m, multi-dose application.
